# On the spectral norms of *r*-circulant matrices with the biperiodic Fibonacci and Lucas numbers

**DOI:** 10.1186/s13660-017-1466-0

**Published:** 2017-08-18

**Authors:** Cahit Köme, Yasin Yazlik

**Affiliations:** Department of Mathematics, Nevşehir Hacı Bektaş Veli University, Nevşehir, 50300 Turkey

**Keywords:** biperiodic Fibonacci number, biperiodic Lucas number, *r*-circulant matrix, norm

## Abstract

In this paper, we present new upper and lower bounds for the spectral norms of the *r*-circulant matrices $Q=C_{r} ( (\frac{b}{a} )^{\frac{\xi (1)}{2}}q_{0}, (\frac{b}{a} )^{\frac{\xi(2)}{2}}q_{1}, (\frac {b}{a} )^{\frac{\xi(3)}{2}}q_{2}, \dots, (\frac{b}{a} )^{\frac{\xi(n)}{2}}q_{n-1} )$ and $L=C_{r} ( (\frac {b}{a} )^{\frac{\xi(0)}{2}}l_{0}, (\frac{b}{a} )^{\frac{\xi (1)}{2}}l_{1}, (\frac{b}{a} )^{\frac{\xi(2)}{2}}l_{2}, \dots, (\frac{b}{a} )^{\frac{\xi(n-1)}{2}}l_{n-1} ) $ whose entries are the biperiodic Fibonacci and biperiodic Lucas numbers, respectively. Finally, we obtain lower and upper bounds for the spectral norms of Kronecker and Hadamard products of *Q* and *L* matrices.

## Introduction

For $n\in\mathbb{N}_{0}$, the Fibonacci and Lucas numbers are defined by $F_{n+2} = F_{n+1} + F_{n}$ and $L_{n+2} = L_{n+1} + L_{n}$ with the initial conditions $F_{0}=0$, $F_{1}=1$ and $L_{0}=2$, $L_{1}=1$, respectively. In recent years, there are several applications and generalizations of Fibonacci and Lucas numbers [1–12]. For example, Falcon and Plaza introduced the *k*-Fibonacci sequence by studying the recursive application of two geometrical transformations used in the well-known 4-triangle longest-edge (4TLE) partition [6]. Edson and Yayenie [3] presented a new generalization of the Fibonacci sequence: for $n\in\mathbb{N}_{0}$,

1 $$ q_{0} = 0, \qquad q_{1}=1, \qquad q_{n+2} = \textstyle\begin{cases} a q_{n+1} + q_{n} & \text{if $n$ is even}, \\ b q_{n+1} + q_{n} & \text{if $n$ is odd}. \end{cases} $$

They also obtained an extended Binet formula for this sequence:

2 $$ q_{n} = \biggl( \frac{a^{1-\xi(n)}}{ab^{\lfloor\frac{n}{2} \rfloor}} \biggr) \frac{\alpha^{n}-\beta^{n}}{\alpha- \beta}, \quad n\in\mathbb{N}_{0}. $$

Afterward, Bilgici [4] defined generalized the Lucas sequence by the following recurrence relation: for $n\in\mathbb{N}_{0}$,

3 $$ l_{0} = 2, \qquad l_{1}=a, \qquad l_{n+2} = \textstyle\begin{cases} b l_{n+1} + l_{n} & \text{if $n$ is even,} \\ a l_{n+1} + l_{n} & \text{if $n$ is odd} \end{cases} $$

and The Binet formula for this sequence is

4 $$ l_{n} = \biggl( \frac{a^{\xi(n)}}{ab^{\lfloor\frac{n+1}{2} \rfloor}} \biggr) \bigl( \alpha^{n}+\beta^{n}\bigr), \quad n\in\mathbb{N}_{0}. $$

In Eqs. (2) and (4), $\alpha= \frac{ab + \sqrt {a^{2}b^{2}+4ab}}{2}$ and $\beta= \frac{ab - \sqrt{a^{2}b^{2}+4ab}}{2}$ are the roots of the characteristic equation of $x^{2}-abx-ab=0$, and $\xi(n) = n- 2\lfloor\frac{n}{2}\rfloor$.

In recent years, there have been several studies on the norms, determinants, and inverses of circulant and *r*-circulant matrices whose entries are special integer sequences [13–27]. For example, Shen and Cen [18] found upper and lower bounds for the spectral norms of *r*-circulant matrices in the forms $A=C_{r}(F_{0}, F_{1}, F_{2}, \dots, F_{n-1})$ and $B=C_{r}(L_{0}, L_{1}, L_{2}, \dots, L_{n-1})$. They also obtained some bounds for the spectral norms of Kronecker and Hadamard products of *A* and *B*. Afterward, Shen and Cen [19] gave the upper and lower bounds for the spectral norms of the matrices $A=C_{r}(F_{k,0}, F_{k,1}, F_{k,2}, \dots , F_{k,n-1})$ and $B=C_{r}(L_{k,0}, L_{k,1}, L_{k,2}, \dots, L_{k,n-1})$. They also presented some bounds for the spectral norms of Hadamard and Kronecker products of these matrices. Bahşi [16] studied the norms of *r*-circulant matrices $H_{r} = \operatorname{Circr}(H_{0}^{(k)},H_{1}^{(k)},H_{2}^{(k)},\dots,H_{n-1}^{(k)})$ and $\widehat{H_{r}} = \operatorname{Circr}(H_{k}^{(0)},H_{k}^{(1)},H_{k}^{(2)},\dots ,H_{k}^{(n-1)})$, where $H_{n}^{(k)}$ denotes the *n*th hyperharmonic number of order *r*.

Inspired by these studies, in this paper, we compute spectral norms of *r*-circulant matrices whose entries are the biperiodic Fibonacci and biperiodic Lucas numbers. This study consists of three sections. The first one is the introduction. In the second section, we give some new theorems, corollaries, and some important results. We give a concise conclusion in the last section.

### Definition 1.1

For any given $c_{0},c_{1},c_{2},\dots,c_{n-1} \in\mathbb{C}$, the *r*-circulant matrix $C_{r} = (c_{ij})_{n\times n}$ is defined by

5 $$ C_{r} = \begin{bmatrix} c_{0} & c_{1} & c_{2} & \ldots& c_{n-2} & c_{n-1} \\ rc_{n-1} & c_{0} & c_{1} & \ldots& c_{n-3} & c_{n-2}\\ rc_{n-2} & rc_{n-1} & c_{0} & \ldots& c_{n-4} & c_{n-3}\\ \vdots & \vdots & \vdots & \ddots & \vdots & \vdots\\ rc_{2} & rc_{3} & rc_{4} & \ldots& c_{0} & c_{1} \\ rc_{1} & rc_{2} & rc_{3} & \ldots& rc_{n-1} & c_{0} \end{bmatrix} . $$

It is clear that, for $r=1$, $C_{r}$ turns into a classical circulant matrix. Let us take any $A=[a_{ij}] \in M_{n,n}(\mathbb{C})$. The Frobenius norm of the matrix *A* is defined by

$$\|A\|_{F} = \Biggl[ \sum_{i=1}^{m} \sum_{j=1}^{n} |a_{ij}|^{2} \Biggr]^{\frac{1}{2}}. $$

Also, the spectral norm of the matrix *A* is given by

$$\|A\|_{2} = \sqrt{\max_{1\leq i\leq n}\lambda_{i} \bigl(A^{H} A\bigr)}, $$

where $\lambda_{i} (A^{H} A)$ are the eigenvalues of $A^{H} A$ such that $A^{H}$ is the conjugate transpose of *A*. Then, the well-known inequality [28] is given by

6 $$ \frac{1}{\sqrt{n}}\|A\|_{F} \leq\|A\|_{2} \leq\|A\|_{F}. $$

### Lemma 1.2

[28]

*For any matrices* $A,B\in M_{m,n}(\mathbb{C})$, *we have*

$$ \|A\circ B\|_{2} \leq\|A\|_{2}\|B\|_{2} , $$

*where* $A\circ B$ *is the Hadamard product of* *A* *and* *B*.

### Lemma 1.3

[28]

*For any matrices* $A\in M_{m,n}(\mathbb{C})$ *and* $B\in M_{p,q}(\mathbb {C})$, *we have*

7 $$ \|A\otimes B\|_{2} = \|A\|_{2} \|B\|_{2}, $$

*where* $A\otimes B$ *is the Kronecker product of* *A* *and* *B*.

### Lemma 1.4

[29]

*For any matrices* $A=[a_{ij}] \in M_{n,n}(\mathbb{C})$ *and* $B=[b_{ij}] \in M_{n,n}(\mathbb{C})$, *we have*

$$ \| A \circ B \|_{2} \leq r_{1}(A)c_{1}(B) , $$

*where* $A \circ B$ *is the Hadamard product*, $r_{1}(A) = \max_{1\leq i \leq n}\sqrt{\sum_{j=1}^{n}|a_{ij}|^{2}}$, *and* $c_{1}(B) = \max_{1\leq j \leq n}\sqrt{\sum_{i=1}^{n}|b_{ij}|^{2}}$.

### Theorem 1.5

[5]

*For any positive integer* *n*, *we have*

8 $$ \sum_{k=1}^{n} \biggl( \frac{b}{a} \biggr)^{\xi(k+1)}q_{k} ^{2} = \biggl( \frac{1}{a} \biggr) q_{n} q_{n+1}. $$

## Main results

In this section, we first give the sum of squares of biperiodic Lucas numbers.

### Theorem 2.1

*For any positive integer* *m*, *we have*

9 $$ \sum_{k=1}^{m} \biggl( \frac{b}{a} \biggr)^{\xi(k)}l_{k} ^{2} = \biggl( \frac {1}{a} \biggr)l_{m+1} l_{m} -2. $$

### Proof

Using the Binet formula of the biperiodic Lucas numbers, we have

$$ \textstyle\begin{cases} l_{k} ^{2} = (\frac{\alpha^{2} }{ab} )^{k} + (\frac{\beta^{2} }{ab} )^{k} + 2 (-1)^{k} & \text{if $k$ is even,} \\ l_{k} ^{2} = ( \frac{a}{b} ) [ (\frac{\alpha^{2} }{ab} )^{k} + (\frac{\beta^{2} }{ab} )^{k} + 2 (-1)^{k} ] & \text{if $k$ is odd}. \end{cases} $$

Therefore, for any $k\geq1$,

$$ \biggl( \frac{b}{a} \biggr)^{\xi(k)}l_{k} ^{2} = \biggl(\frac{\alpha^{2} }{ab} \biggr)^{k} + \biggl(\frac{\beta^{2} }{ab} \biggr)^{k} + 2 (-1)^{k}. $$

Using the properties $ab(\alpha+1)=\alpha^{2}$ and $ab(\beta+1)=\beta ^{2}$, we get

$$ \begin{aligned} \sum_{k=1}^{m} \biggl( \frac{b}{a} \biggr)^{\xi(k)}l_{k} ^{2} &= \sum_{k=1}^{m} \biggl(\frac{\alpha^{2} }{ab} \biggr)^{k} + \sum_{k=1}^{m} \biggl(\frac{\beta^{2} }{ab} \biggr)^{k} + \sum _{k=1}^{m} 2 (-1)^{k} \\ & = \frac{ (\frac{\alpha^{2}}{ab} )^{m+1} - (\frac{\alpha ^{2}}{ab} )}{ (\frac{\alpha^{2}}{ab} ) -1 } + \frac{ (\frac{\beta^{2}}{ab} )^{m+1} - (\frac{\beta^{2}}{ab} )}{ (\frac{\beta^{2}}{ab} ) -1 } + (-1)^{m} - 1 \\ & = \frac{1}{(ab)^{m+1}} \bigl[ \alpha^{2m+1} + \beta^{2m+1} - (-1)^{m} \bigr] -2. \end{aligned} $$

Observe that

$$ \biggl(\frac{1}{a} \biggr)l_{m} l_{m+1} = \frac{1}{(ab)^{m+1}} \bigl[ \alpha ^{2m+1} + \beta^{2m+1} - (-1)^{m} \bigr]. $$

Therefore,

$$ \sum_{k=1}^{m} \biggl( \frac{b}{a} \biggr)^{\xi(k)}l_{k} ^{2} = \biggl(\frac {1}{a} \biggr)l_{m+1} l_{m} -2. $$

 □

### Theorem 2.2

*Let* $Q=C_{r} ( (\frac{b}{a} )^{\frac{\xi(1)}{2}}q_{0}, (\frac{b}{a} )^{\frac{\xi(2)}{2}}q_{1}, (\frac{b}{a} )^{\frac{\xi(3)}{2}}q_{2}, \dots, (\frac{b}{a} )^{\frac{\xi (n)}{2}}q_{n-1} ) $ *be an* *r*-*circulant matrix*. *Then*, *for* $r \in \mathbb{C}$, *we have*:

- *if* $|r| \geq1$, *then*
  $$\sqrt{\frac{q_{n} q_{n-1}}{a}} \leq\|Q\|_{2} \leq|r|\frac{q_{n} q_{n-1}}{a}; $$
- *if* $|r| < 1$, *then*
  $$|r|\sqrt{\frac{q_{n} q_{n-1}}{a}} \leq\|Q\|_{2} \leq\sqrt{(n-1) \frac{q_{n} q_{n-1}}{a}}. $$

### Proof

The matrix *Q* is of the form

10 $$ Q = \begin{bmatrix} ( \frac{b}{a} )^{\frac{\xi(1)}{2}}q_{0} & ( \frac {b}{a} )^{\frac{\xi(2)}{2}}q_{1} & ( \frac{b}{a} )^{\frac {\xi{(3)}}{2}} q_{2} & \ldots& ( \frac{b}{a} )^{\frac{\xi {(n)}}{2}} q_{n-1} \\ r ( \frac{b}{a} )^{\frac{\xi{(n)}}{2}} q_{n-1} & ( \frac {b}{a} )^{\frac{\xi{(1)}}{2}} q_{0} & ( \frac{b}{a} )^{\frac{\xi{(2)}}{2}} q_{1} & \ldots& ( \frac{b}{a} )^{\frac {\xi{(n-1)}}{2}} q_{n-2}\\ r ( \frac{b}{a} )^{\frac{\xi{(n-1)}}{2}} q_{n-2} & r ( \frac{b}{a} )^{\frac{\xi{(n)}}{2}} q_{n-1} & ( \frac {b}{a} )^{\frac{\xi{(1)}}{2}} q_{0} & \ldots& ( \frac {b}{a} )^{\frac{\xi{(n-2)}}{2}} q_{n-3}\\ \vdots& \vdots& \vdots& \ddots& \vdots\\ r ( \frac{b}{a} )^{\frac{\xi{(2)}}{2}} q_{1} & r ( \frac {b}{a} )^{\frac{\xi{(3)}}{2}} q_{2} & r ( \frac{b}{a} )^{\frac{\xi{(4)}}{2}} q_{3} & \ldots& ( \frac{b}{a} )^{\frac {\xi{(1)}}{2}} q_{0} \end{bmatrix} . $$

Then we have

$$ \|Q\|_{F}^{2} = \sum_{k=0}^{n-1}(n-k) \biggl( \frac{b}{a} \biggr)^{\xi {(k+1)}} q_{k}^{2} + \sum_{k=1}^{n-1} k|r|^{2} \biggl( \frac{b}{a} \biggr)^{\xi{(k+1)}} q_{k}^{2}. $$

Hence, for $|r|\geq1$, using Eq. (8), we obtain

$$ \begin{aligned} \|Q\|_{F}^{2} &\geq\sum _{k=0}^{n-1}(n-k) \biggl( \frac{b}{a} \biggr)^{\xi{(k+1)}} q_{k}^{2} + \sum _{k=1}^{n-1} k \biggl( \frac{b}{a} \biggr)^{\xi{(k+1)}} q_{k}^{2} \\ & = n \sum_{k=0}^{n-1} \biggl( \frac{b}{a} \biggr)^{\xi{(k+1)}} q_{k}^{2} \\ & = n \biggl( \frac{q_{n} q_{n-1}}{a} \biggr), \end{aligned} $$

that is,

$$ \frac{1}{\sqrt{n}}\|Q\|_{F} \geq\sqrt{\frac{q_{n} q_{n-1}}{a}}. $$

From (6) we have

$$ \|Q\|_{2} \geq\sqrt{\frac{q_{n} q_{n-1}}{a}}. $$

Now, for $|r|\geq1$, we give an bound for the spectral norm of the matrix *Q*. Let the matrices *B* and *C* be

11 $$ B = \begin{bmatrix} r ( \frac{b}{a} )^{\frac{\xi{(1)}}{2}} q_{0} & 1 & 1 & \ldots& 1 \\ r ( \frac{b}{a} )^{\frac{\xi{(n)}}{2}} q_{n-1} & r ( \frac{b}{a} )^{\frac{\xi{(1)}}{2}} q_{0} & 1 & \ldots& 1\\ r ( \frac{b}{a} )^{\frac{\xi{(n-1)}}{2}} q_{n-2} & r ( \frac{b}{a} )^{\frac{\xi{(n)}}{2}} q_{n-1} & r ( \frac {b}{a} )^{\frac{\xi{(1)}}{2}} q_{0} & \ldots& 1\\ \vdots& \vdots& \vdots& \ddots& \vdots\\ r ( \frac{b}{a} )^{\frac{\xi{(2)}}{2}} q_{1} & r ( \frac {b}{a} )^{\frac{\xi{(3)}}{2}} q_{2} & r ( \frac{b}{a} )^{\frac{\xi{(4)}}{2}} q_{3} & \ldots& r ( \frac{b}{a} )^{\frac {\xi{(1)}}{2}} q_{0} \end{bmatrix} $$

and

12 $$ C = \begin{bmatrix} ( \frac{b}{a} )^{\frac{\xi{(1)}}{2}} q_{0} & ( \frac {b}{a} )^{\frac{\xi{(2)}}{2}} q_{1} & ( \frac{b}{a} )^{\frac{\xi{(3)}}{2}} q_{2} & \ldots& ( \frac{b}{a} )^{\frac {\xi{(n)}}{2}} q_{n-1} \\ 1 & ( \frac{b}{a} )^{\frac{\xi{(1)}}{2}} q_{0} & ( \frac {b}{a} )^{\frac{\xi{(2)}}{2}} q_{1} & \ldots& ( \frac {b}{a} )^{\frac{\xi{(n-1)}}{2}} q_{n-2}\\ 1 & 1 & ( \frac{b}{a} )^{\frac{\xi{(1)}}{2}} q_{0} & \ldots& ( \frac{b}{a} )^{\frac{\xi{(n-2)}}{2}} q_{n-3}\\ \vdots& \vdots& \vdots& \ddots& \vdots\\ 1 & 1 & 1 & \ldots& ( \frac{b}{a} )^{\frac{\xi{(1)}}{2}} q_{0} \end{bmatrix} , $$

so that $Q = B \circ C$. Then we obtain

$$\begin{gathered} r_{1}(B) = \max_{1\leq i\leq n} \sqrt{\sum _{j=1}^{n}|b_{ij}|^{2} } = \sqrt {|r|^{2}\sum_{k=0}^{n-1} \biggl( \frac{b}{a} \biggr)^{\xi(k+1)} q_{k}^{2} } = |r|\sqrt{\frac{q_{n} q_{n-1}}{a}}, \\c_{1}(C) = \max_{1\leq j \leq n} \sqrt{\sum _{i=1}^{n}|c_{ij}|^{2} } = \sqrt {\sum_{k=0}^{n-1} \biggl( \frac{b}{a} \biggr)^{\xi(k+1)} q_{k}^{2} } = \sqrt {\frac{q_{n} q_{n-1}}{a}}. \end{gathered}$$

By Lemma 1.4 we have

$$ \|Q\|_{2} \leq r_{1}(B)c_{1}(C) = |r| \frac{q_{n} q_{n-1}}{a}. $$

Thus,

$$ \sqrt{\frac{q_{n} q_{n-1}}{a}} \leq\|Q\|_{2} \leq|r|\frac{q_{n} q_{n-1}}{a}. $$

On the other hand, for $|r|<1$, we have

$$ \begin{aligned} \|Q\|_{F}^{2} &\geq\sum _{k=0}^{n-1}(n-k) |r|^{2} \biggl( \frac {b}{a} \biggr)^{\xi{(k+1)}} q_{k}^{2} + \sum _{k=1}^{n-1} k|r|^{2} \biggl( \frac{b}{a} \biggr)^{\xi{(k+1)}} q_{k}^{2} \\ &= n|r|^{2}\sum_{k=0}^{n-1} \biggl( \frac{b}{a} \biggr)^{\xi{(k+1)}} q_{k}^{2} \\ & = n|r|^{2} \biggl( \frac{q_{n} q_{n-1}}{a} \biggr), \end{aligned} $$

that is,

$$ \frac{1}{\sqrt{n}}\|Q\|_{F}\geq|r|\sqrt{\frac{q_{n} q_{n-1}}{a}}. $$

Thus, we obtain

$$ \|Q\|_{2} \geq|r|\sqrt{\frac{q_{n} q_{n-1}}{a}}. $$

Now, for $|r|< 1$, we give an upper bound for the spectral norm of the matrix *Q*. Let the matrices *D* and *E* be

13 $$ D = \begin{bmatrix} ( \frac{b}{a} )^{\frac{\xi{(1)}}{2}} q_{0} & 1 & 1 & \ldots& 1 \\ r & ( \frac{b}{a} )^{\frac{\xi{(1)}}{2}} q_{0} & 1 & \ldots& 1\\ r & r & ( \frac{b}{a} )^{\frac{\xi{(1)}}{2}} q_{0} & \ldots& 1\\ \vdots& \vdots& \vdots& \ddots& \vdots\\ r & r & r & \ldots& ( \frac{b}{a} )^{\frac{\xi{(1)}}{2}} q_{0} \end{bmatrix} $$

and

14 $$ E = \begin{bmatrix} ( \frac{b}{a} )^{\frac{\xi{(1)}}{2}} q_{0} & ( \frac {b}{a} )^{\frac{\xi{(2)}}{2}} q_{1} & ( \frac{b}{a} )^{\frac{\xi{(3)}}{2}} q_{2} & \ldots& ( \frac{b}{a} )^{\frac {\xi{(n)}}{2}} q_{n-1} \\ ( \frac{b}{a} )^{\frac{\xi{(n)}}{2}} q_{n-1} & ( \frac {b}{a} )^{\frac{\xi{(1)}}{2}} q_{0} & ( \frac{b}{a} )^{\frac{\xi{(2)}}{2}} q_{1} & \ldots& ( \frac{b}{a} )^{\frac {\xi{(n-1)}}{2}} q_{n-2}\\ ( \frac{b}{a} )^{\frac{\xi{(n-1)}}{2}} q_{n-2} & ( \frac {b}{a} )^{\frac{\xi{(n)}}{2}} q_{n-1} & ( \frac{b}{a} )^{\frac{\xi{(1)}}{2}} q_{0} & \ldots& ( \frac{b}{a} )^{\frac {\xi{(n-2)}}{2}} q_{n-3}\\ \vdots& \vdots& \vdots& \ddots& \vdots\\ ( \frac{b}{a} )^{\frac{\xi{(2)}}{2}} q_{1} & ( \frac {b}{a} )^{\frac{\xi{(3)}}{2}} q_{2} & ( \frac{b}{a} )^{\frac{\xi{(4)}}{2}} q_{3} & \ldots& ( \frac{b}{a} )^{\frac {\xi{(1)}}{2}} q_{0} \end{bmatrix} , $$

so that $Q=D \circ E$. Then we obtain

$$\begin{gathered} r_{1}(D) = \max_{1\leq i\leq n} \sqrt{\sum _{j=1}^{n}|d_{ij}|^{2} } = \sqrt { \biggl( \frac{b}{a} \biggr)^{\frac{\xi(1)}{2}} q_{0} ^{2} + (n-1)} = \sqrt{n-1}, \\c_{1}(E) = \max_{1\leq j \leq n} \sqrt{\sum _{i=1}^{n}|e_{ij}|^{2} } = \sqrt {\sum_{k=0}^{n-1} \biggl( \frac{b}{a} \biggr)^{\xi(k+1)} q_{k}^{2} } = \sqrt {\frac{q_{n} q_{n-1}}{a}}. \end{gathered}$$

By Lemma 1.4 we have

$$ \|Q\|_{2} \leq r_{1}(D)c_{1}(E) = \sqrt{(n-1) \frac{q_{n} q_{n-1}}{a}}. $$

Thus,

$$ |r|\sqrt{\frac{q_{n} q_{n-1}}{a}} \leq\|Q\|_{2} \leq\sqrt{(n-1) \frac {q_{n} q_{n-1}}{a}}. $$

 □

### Theorem 2.3

*Let* $L=C_{r} ( (\frac{b}{a} )^{\frac{\xi(0)}{2}}l_{0}, (\frac{b}{a} )^{\frac{\xi(1)}{2}}l_{1}, (\frac{b}{a} )^{\frac{\xi(2)}{2}}l_{2}, \dots, (\frac{b}{a} )^{\frac{\xi (n-1)}{2}}l_{n-1} ) $ *be an* *r*-*circulant matrix*. *Then*, *for* $r \in\mathbb{C}$, *we have*:

- *if* $|r| \geq1$, *then*
  $$\sqrt{\frac{l_{n} l_{n-1}}{a}+2} \leq\|L\|_{2} \leq|r| \biggl( \frac{l_{n} l_{n-1}}{a}+2 \biggr); $$
- *if* $|r| < 1$, *then*
  $$|r|\sqrt{\frac{l_{n} l_{n-1}}{a}+2} \leq\|L\|_{2} \leq\sqrt{n \biggl( \frac {l_{n} l_{n-1}}{a}+2 \biggr)}. $$

### Proof

The matrix *L* is of the form

15 $$ L = \begin{bmatrix} ( \frac{b}{a} )^{\frac{\xi(0)}{2}}l_{0} & ( \frac {b}{a} )^{\frac{\xi(1)}{2}}l_{1} & ( \frac{b}{a} )^{\frac {\xi{(2)}}{2}} l_{2} & \ldots& ( \frac{b}{a} )^{\frac{\xi {(n-1)}}{2}} l_{n-1} \\ r ( \frac{b}{a} )^{\frac{\xi{(n-1)}}{2}} l_{n-1} & ( \frac{b}{a} )^{\frac{\xi{(0)}}{2}} l_{0} & ( \frac{b}{a} )^{\frac{\xi{(1)}}{2}} l_{1} & \ldots& ( \frac{b}{a} )^{\frac {\xi{(n-2)}}{2}} l_{n-2}\\ r ( \frac{b}{a} )^{\frac{\xi{(n-2)}}{2}} l_{n-2} & r ( \frac{b}{a} )^{\frac{\xi{(n-1)}}{2}} l_{n-1} & ( \frac {b}{a} )^{\frac{\xi{(0)}}{2}} l_{0} & \ldots& ( \frac {b}{a} )^{\frac{\xi{(n-3)}}{2}} l_{n-3}\\ \vdots& \vdots& \vdots& \ddots& \vdots\\ r ( \frac{b}{a} )^{\frac{\xi{(1)}}{2}} l_{1} & r ( \frac {b}{a} )^{\frac{\xi{(2)}}{2}} l_{2} & r ( \frac{b}{a} )^{\frac{\xi{(3)}}{2}} l_{3} & \ldots& ( \frac{b}{a} )^{\frac {\xi{(0)}}{2}} l_{0} \end{bmatrix} . $$

Then we have

$$ \|L\|_{F}^{2} = \sum_{k=0}^{n-1}(n-k) \biggl( \frac{b}{a} \biggr)^{\xi {(k)}} l_{k}^{2} + \sum_{k=1}^{n-1} k|r|^{2} \biggl( \frac{b}{a} \biggr)^{\xi{(k)}} l_{k}^{2}. $$

Hence, for $|r|\geq1$, using Eq. (9), we obtain

$$ \begin{aligned} \|L\|_{F}^{2} &\geq\sum _{k=0}^{n-1}(n-k) \biggl( \frac{b}{a} \biggr)^{\xi{(k)}} l_{k}^{2} + \sum _{k=1}^{n-1} k \biggl( \frac{b}{a} \biggr)^{\xi{(k)}} l_{k}^{2} \\ & = n \sum_{k=0}^{n-1} \biggl( \frac{b}{a} \biggr)^{\xi{(k)}} l_{k}^{2} \\ & = n \biggl( \frac{l_{n} l_{n-1}}{a} +2 \biggr), \end{aligned} $$

that is,

$$ \frac{1}{\sqrt{n}}\|L\|_{F} \geq\sqrt{\frac{l_{n} l_{n-1}}{a}+2}. $$

From (6) we have

$$ \|L\|_{2} \geq\sqrt{\frac{l_{n} l_{n-1}}{a}+2}. $$

Now, for $|r|\geq1$, we give an upper bound for the spectral norm of the matrix *L*. Let the matrices *F* and *H* be

16 $$ F = \begin{bmatrix} r ( \frac{b}{a} )^{\frac{\xi{(0)}}{2}} l_{0} & 1 & 1 & \ldots& 1 \\ r ( \frac{b}{a} )^{\frac{\xi{(n-1)}}{2}} l_{n-1} & r ( \frac{b}{a} )^{\frac{\xi{(0)}}{2}} l_{0} & 1 & \ldots& 1\\ r ( \frac{b}{a} )^{\frac{\xi{(n-2)}}{2}} l_{n-2} & r ( \frac{b}{a} )^{\frac{\xi{(n-1)}}{2}} l_{n-1} & r ( \frac {b}{a} )^{\frac{\xi{(0)}}{2}} l_{0} & \ldots& 1\\ \vdots& \vdots& \vdots& \ddots& \vdots\\ r ( \frac{b}{a} )^{\frac{\xi{(1)}}{2}} l_{1} & r ( \frac {b}{a} )^{\frac{\xi{(2)}}{2}} l_{2} & r ( \frac{b}{a} )^{\frac{\xi{(3)}}{2}} l_{3} & \ldots& r ( \frac{b}{a} )^{\frac {\xi{(0)}}{2}} l_{0} \end{bmatrix} $$

and

17 $$ H = \begin{bmatrix} ( \frac{b}{a} )^{\frac{\xi{(0)}}{2}} l_{0} & ( \frac {b}{a} )^{\frac{\xi{(1)}}{2}} l_{1} & ( \frac{b}{a} )^{\frac{\xi{(2)}}{2}} l_{2} & \ldots& ( \frac{b}{a} )^{\frac {\xi{(n-1)}}{2}} l_{n-1} \\ 1 & ( \frac{b}{a} )^{\frac{\xi{(0)}}{2}} l_{0} & ( \frac {b}{a} )^{\frac{\xi{(1)}}{2}} l_{1} & \ldots& ( \frac {b}{a} )^{\frac{\xi{(n-2)}}{2}} l_{n-2}\\ 1 & 1 & ( \frac{b}{a} )^{\frac{\xi{(0)}}{2}} l_{0} & \ldots& ( \frac{b}{a} )^{\frac{\xi{(n-3)}}{2}} l_{n-3}\\ \vdots& \vdots& \vdots& \ddots& \vdots\\ 1 & 1 & 1 & \ldots& ( \frac{b}{a} )^{\frac{\xi{(0)}}{2}} l_{0} \end{bmatrix} , $$

so that $L = F \circ H$. Then we obtain

$$\begin{gathered} r_{1}(F) = \max_{1\leq i\leq n} \sqrt{\sum _{j=1}^{n}|f_{ij}|^{2} } = \sqrt {|r|^{2}\sum_{k=0}^{n-1} \biggl( \frac{b}{a} \biggr)^{\xi(k)} l_{k}^{2} } = |r|\sqrt{\frac{l_{n} l_{n-1}}{a}+2}, \\c_{1}(H) = \max_{1\leq j \leq n} \sqrt{\sum _{i=1}^{n}|h_{ij}|^{2} } = \sqrt {\sum_{k=0}^{n-1} \biggl( \frac{b}{a} \biggr)^{\xi(k)} l_{k}^{2} } = \sqrt {\frac{l_{n} l_{n-1}}{a}+2}. \end{gathered}$$

By Lemma 1.4 we have

$$ \|L\|_{2} \leq r_{1}(F)c_{1}(H) = |r| \biggl( \frac{l_{n} l_{n-1}}{a} + 2 \biggr). $$

Thus,

$$ \sqrt{\frac{l_{n} l_{n-1}}{a}+2} \leq\|L\|_{2} \leq|r| \biggl( \frac{l_{n} l_{n-1}}{a} + 2 \biggr). $$

On the other hand, for $|r|<1$, we have

$$ \begin{aligned} \|L\|_{F}^{2} &\geq\sum _{k=0}^{n-1}(n-k) |r|^{2} \biggl( \frac {b}{a} \biggr)^{\xi{(k)}} l_{k}^{2} + \sum _{k=1}^{n-1} k|r|^{2} \biggl( \frac{b}{a} \biggr)^{\xi{(k)}} l_{k}^{2} \\ &= n|r|^{2}\sum_{k=0}^{n-1} \biggl( \frac{b}{a} \biggr)^{\xi{(k)}} l_{k}^{2} \\ & = n|r|^{2} \biggl( \frac{l_{n} l_{n-1}}{a} +2 \biggr), \end{aligned} $$

that is,

$$ \frac{1}{\sqrt{n}}\|L\|_{F}\geq|r|\sqrt{\frac{l_{n} l_{n-1}}{a}+2}. $$

Thus, we obtain

$$ \|L\|_{2} \geq|r|\sqrt{\frac{l_{n} l_{n-1}}{a}+2}. $$

Now, for $|r|< 1$, we give an upper bound for the spectral norm of the matrix *L*. Let the matrices *G* and *K* be

18 $$ G = \begin{bmatrix} 1 & 1 & 1 & \ldots& 1 \\ r & 1 & 1 & \ldots& 1\\ r & r & 1 & \ldots& 1\\ \vdots & \vdots & \vdots & \ddots & \vdots\\ r & r & r & \ldots& 1 \end{bmatrix} $$

and

19 $$ K = \begin{bmatrix} ( \frac{b}{a} )^{\frac{\xi(0)}{2}}l_{0} & ( \frac {b}{a} )^{\frac{\xi(1)}{2}}l_{1} & ( \frac{b}{a} )^{\frac {\xi{(2)}}{2}} l_{2} & \ldots& ( \frac{b}{a} )^{\frac{\xi {(n-1)}}{2}} l_{n-1} \\ ( \frac{b}{a} )^{\frac{\xi{(n-1)}}{2}} l_{n-1} & ( \frac {b}{a} )^{\frac{\xi{(0)}}{2}} l_{0} & ( \frac{b}{a} )^{\frac{\xi{(1)}}{2}} l_{1} & \ldots& ( \frac{b}{a} )^{\frac {\xi{(n-2)}}{2}} l_{n-2}\\ ( \frac{b}{a} )^{\frac{\xi{(n-2)}}{2}} l_{n-2} & ( \frac {b}{a} )^{\frac{\xi{(n-1)}}{2}} l_{n-1} & ( \frac{b}{a} )^{\frac{\xi{(0)}}{2}} l_{0} & \ldots& ( \frac{b}{a} )^{\frac {\xi{(n-3)}}{2}} l_{n-3}\\ \vdots& \vdots& \vdots& \ddots& \vdots\\ ( \frac{b}{a} )^{\frac{\xi{(1)}}{2}} l_{1} & ( \frac {b}{a} )^{\frac{\xi{(2)}}{2}} l_{2} & ( \frac{b}{a} )^{\frac{\xi{(3)}}{2}} l_{3} & \ldots& ( \frac{b}{a} )^{\frac {\xi{(0)}}{2}} l_{0} \end{bmatrix} , $$

so that $L=G \circ K$. Then we obtain

$$\begin{gathered} r_{1}(G) = \max_{1\leq i\leq n} \sqrt{\sum _{j=1}^{n}|g_{ij}|^{2} } = \sqrt{n}, \\c_{1}(K) = \max_{1\leq j \leq n} \sqrt{\sum _{i=1}^{n}|k_{ij}|^{2} } = \sqrt {\sum_{k=0}^{n-1} \biggl( \frac{b}{a} \biggr)^{\xi(k)} l_{k}^{2} } = \sqrt {\frac{l_{n} l_{n-1}}{a}+2}. \end{gathered}$$

By Lemma 1.4 we have

$$ \|L\|_{2} \leq r_{1}(G)c_{1}(K) = \sqrt{n \biggl(\frac{l_{n} l_{n-1}}{a} +2 \biggr)}. $$

Thus,

$$ |r|\sqrt{\frac{l_{n} l_{n-1}}{a}+2} \leq\|L\|_{2} \leq\sqrt{n \biggl( \frac {l_{n} l_{n-1}}{a}+2 \biggr)}. $$

 □

### Corollary 2.1

*Let* $Q=C_{r} ( (\frac{b}{a} )^{\frac{\xi(1)}{2}}q_{0}, (\frac{b}{a} )^{\frac{\xi(2)}{2}}q_{1}, (\frac{b}{a} )^{\frac{\xi(3)}{2}}q_{2}, \dots, (\frac{b}{a} )^{\frac{\xi (n)}{2}}q_{n-1} )$ *and* $L= C_{r} ( (\frac{b}{a} )^{\frac{\xi(0)}{2}}l_{0}, (\frac{b}{a} )^{\frac{\xi (1)}{2}}l_{1}, (\frac{b}{a} )^{\frac{\xi(2)}{2}}l_{2}, \dots, (\frac{b}{a} )^{\frac{\xi(n-1)}{2}}l_{n-1} ) $ *be* *r*-*circulant matrices*, *where* $r\in\mathbb{C}$.

(i) *If* $|r|\geq1$, *then*
  $$ \|Q\circ L\|_{2} \leq|r|^{2} \frac{q_{n}q_{n-1}}{a} \biggl( \frac{l_{n} l_{n-1}}{a} + 2 \biggr). $$
(ii) *If* $|r| < 1$, *then*
  $$ \|Q\circ L\|_{2} \leq\sqrt{n(n-1)\frac{q_{n}q_{n-1}}{a} \biggl( \frac{l_{n} l_{n-1}}{a} +2 \biggr)}. $$

### Proof

Since $\|Q\circ L\|_{2} \leq\|Q\|_{2} \|L\|_{2}$, the proof is trivial by Theorems 2.2 and 2.3. □

### Corollary 2.2

*Let* $Q=C_{r} ( (\frac{b}{a} )^{\frac{\xi(1)}{2}}q_{0}, (\frac{b}{a} )^{\frac{\xi(2)}{2}}q_{1}, (\frac{b}{a} )^{\frac{\xi(3)}{2}}q_{2}, \dots, (\frac{b}{a} )^{\frac{\xi (n)}{2}}q_{n-1} )$ *and* $L= C_{r} ( (\frac{b}{a} )^{\frac{\xi(0)}{2}}l_{0}, (\frac{b}{a} )^{\frac{\xi (1)}{2}}l_{1}, (\frac{b}{a} )^{\frac{\xi(2)}{2}}l_{2}, \dots, (\frac{b}{a} )^{\frac{\xi(n-1)}{2}}l_{n-1} ) $ *be* *r*-*circulant matrices*, *where* $r\in\mathbb{C}$.

(i) *If* $|r|\geq1$, *then*
  $$ \|Q \otimes L\|_{2} \geq\sqrt{\frac{q_{n}q_{n-1}}{a} \biggl( \frac{l_{n} l_{n-1}}{a} + 2 \biggr)} $$
  *and*
  $$ \|Q\otimes L\|_{2} \leq|r|^{2} \frac{q_{n}q_{n-1}}{a} \biggl( \frac{l_{n} l_{n-1}}{a} + 2 \biggr)l. $$
(ii) *If* $|r|< 1$, *then*
  $$ \|Q \otimes L\|_{2} \geq|r|^{2} \sqrt{\frac{q_{n} q_{n-1}}{a} \biggl( \frac {l_{n} l_{n-1}}{a} + 2 \biggr)} $$
  *and*
  $$ \|Q\otimes L\|_{2} \leq\sqrt{n(n-1)\frac{q_{n}q_{n-1}}{a} \biggl( \frac {l_{n} l_{n-1}}{a} +2 \biggr)}. $$

### Proof

Since $\|Q\otimes L\|_{2} = \|Q\|_{2}\|L\|_{2}$, the proof is trivial by Theorems 2.2 and 2.3. □

## Conclusion

In this paper, we obtain new upper and lower bounds for the spectral norms of the *r*-circulant matrices *Q* and *L* whose entries are the biperiodic Fibonacci and biperiodic Lucas numbers. This study can be reduced to various studies for the specific values of *a* and *b* in the literature. For example, if $a=b=r=1$, $a=b=1$, and $a=b=k$ in *Q* and *L*, our results reduce to the studies [13, 18], and [19], respectively. Since this study is a generalization of these studies, it contributes to the literature by providing essential information on the spectral norms of *r*-circulant matrices.
