# Correction to: On the spectral norms of *r*-circulant matrices with the bi-periodic Fibonacci and Lucas numbers

**DOI:** 10.1186/s13660-018-1642-x

**Published:** 2018-02-22

**Authors:** Cahit Köme, Yasin Yazlik

**Affiliations:** Department of Mathematics, Nevşehir Hacı Bektaş Veli University, Nevşehir, Turkey

## Correction

In the publication of this article [1], there are a few errors.

1. Page 4, line 4:
  The statement $\frac{1}{(ab)^{m+1}} [ \alpha^{2m+1} + \beta^{2m+1} - (-1)^{m} ] -2$ should instead read: $\frac{1}{(ab)^{m+1}} [ \alpha^{2m+1} + \beta^{2m+1} ] + (-1)^{m} -2$.
2. Page 4, line 6:
  The statement $( \frac{1}{a} ) l_{m} l_{m+1} = \frac{1}{(ab)^{m+1}} [ \alpha^{2m+1} + \beta^{2m+1} - (-1)^{m} ] $ should instead read: $( \frac{1}{a} ) l _{m} l_{m+1} = \frac{1}{(ab)^{m+1}} [ \alpha^{2m+1} + \beta^{2m+1} ] + (-1)^{m}$.
3. Page 8, Equation (16):
  The matrix *F* should instead read:
  $$ F = \begin{bmatrix} 1 & 1 & 1 & \ldots & 1 \\ r ( \frac{b}{a} ) ^{\frac{\xi {(n-1)}}{2}} l_{n-1} & 1 & 1 & \ldots & 1 \\ r ( \frac{b}{a} ) ^{\frac{\xi {(n-2)}}{2}} l_{n-2} & r ( \frac{b}{a} ) ^{\frac{\xi {(n-1)}}{2}} l_{n-1} & 1 & \ldots & 1 \\ \vdots & \vdots & \vdots & \ddots & \vdots \\ r ( \frac{b}{a} ) ^{\frac{\xi {(1)}}{2}} l_{1} & r ( \frac{b}{a} ) ^{\frac{\xi {(2)}}{2}} l_{2} & r ( \frac{b}{a} ) ^{\frac{\xi {(3)}}{2}} l_{3} & \ldots & 1 \end{bmatrix} . $$
4. Page 8, line 15: The equation $r_{1}(F)$ should instead read:
  $$ r_{1}(F) = \max_{1\leq i\leq n} \sqrt{\sum _{j=1}^{n}\vert f_{ij} \vert ^{2} } = \sqrt{1 + \vert r \vert ^{2}\sum _{k=1}^{n-1} \biggl( \frac{b}{a} \biggr) ^{\xi (k)} l_{k}^{2} } = \sqrt{1 + \vert r \vert ^{2} \biggl( \frac{l_{n} l_{n-1}}{a}-2 \biggr) }. $$
5. Page 9, lines 2 and 4, page 10, line 11 and Theorem 2.3 on page 7:
  The statement $\vert r \vert ( \frac{l_{n} l_{n-1}}{a} + 2 ) $ should instead read:
  $$ \sqrt{\frac{l_{n} l_{n-1}}{a}+2} \sqrt{1 + \vert r \vert ^{2} \biggl( \frac{l _{n} l_{n-1}}{a}-2 \biggr) }. $$
6. Page 10, line 20:
  The statement $\vert r \vert ^{2} \frac{q_{n}q_{n-1}}{a} ( \frac{l_{n} l _{n-1}}{a} + 2 ) l$ should instead read:
  $$ \vert r \vert \frac{q_{n}q_{n-1}}{a} \sqrt{\frac{l_{n} l_{n-1}}{a}+2} \sqrt{1 + \vert r \vert ^{2} \biggl( \frac{l_{n} l_{n-1}}{a}-2 \biggr) }. $$

This has now been included in this erratum.
